# Parenting Stress and Parenting Efficacy of Parents Having Children with Disabilities in China: The Role of Social Support

**DOI:** 10.3390/ijerph20032133

**Published:** 2023-01-24

**Authors:** Wangqian Fu, Rui Li, Yaqian Zhang, Ke Huang

**Affiliations:** 1 School of Special Education, Beijing Normal University, Beijing 100875, China; 2 School of Education, Beijing Sport University, Beijing 100091, China; 3 Faculty of Education, Beijing Normal University, Beijing 100875, China; 4 Alum Rock Union School District, San Jose, CA 95127, USA

**Keywords:** parenting stress, parenting efficacy, social support, disabled children

## Abstract

Raising children with disabilities is challenging for parents, who experience high parenting stress. The study aimed to understand the status quo of parenting efficacy of parents having children with disabilities and to analyze the association between parent efficacy, parenting stress, and social support in China. We surveyed 373 parents having children with disabilities enrolled in special education schools or rehabilitation institutions from 14 provinces in China. The online questionnaire including Parental Stress Index-Short Form, the Child Adjustment and Parent Efficacy Scale-Developmental Disability (CAPES-DD), and Social Support Scale was applied in the study. The results suggested that parenting efficacy of parents having children with disabilities in China was at the medium level. Parenting stress, social support, and parenting efficacy were significantly related with each other, and social support played a mediating role between parenting stress and parenting efficacy. The findings indicated that reducing parenting stress and improving social support might improve parenting efficacy. We also discussed the implications of providing intervention strategies or social support to improve parenting efficacy for parents with disabled children in China.

## 1. Introduction

Parenting stress refers to a series of processes that result in repugnant psychological and physical reactions to trying to fit in with the demands of parenting [1]. It is a special kind of pressure that parents feel when they take on parental responsibilities [2]. Parents of children with developmental disabilities (e.g., intellectual disability, autism, cerebral palsy, visual impairment, hearing impairment, ADHD) tend to experience higher parenting stress than others [3,4,5,6,7,8].

Parenting stress can negatively affect children’s development, directly or indirectly [9]. It is directly influenced by the behavioral factors in children [10,11]. For parents experiencing high level parenting stress may impair their perceptions of the disability and their responses to children, thereby affecting their child’s skill development [12]. For example, children with autism exhibit more behavioral problems than children without autism; parents of children with autism experienced a higher level of parenting stress [13]. Moreover, parenting stress is also closely related with the severity of disability of children [14,15]. Parents of children with disabilities experiencing high parenting stress may suffer psychological distress or other mental illness [16,17].

Parenting efficacy comes mostly from the concept of self-efficacy [18]. Based on social cognition theory [19], parenting efficacy is an individual’s judgment and cognition of his or her ability to influence the child’s development and environment in parenting practices [20]. Parenting efficacy has been considered a major determinant of parenting behavior and is strongly associated with child development outcomes and child psychological adjustment [21,22,23]. Parents with higher parenting efficacy believe that they have good parenting ability, can complete parenting tasks, and have positive influence on children’s development [9]. In particular, increased parenting efficacy can significantly improve the development of children with disability [24]. Parenting efficacy has a close relationship with parenting stress, since they have the same empirical background, and parenting stress may alter parenting efficacy over time [9]. Although the relationship between parenting stress and parenting efficacy has been established, the underlying mechanisms between parenting stress and parenting efficacy for parents of children with disabilities were still unclear.

Social support refers to an individual’s perception or experience of being cared for, respected, and included in a mutually supportive social network that benefits physical and mental health [25,26]. Empirical studies have found that there is a significant negative correlation between parenting stress and social support for parents of children with disabilities [27,28,29,30,31,32]. During the COVID-19 pandemic, less social support, and psychological and behavioral problems in parents with disabled children were significantly related with higher levels of parenting stress [33].

Social support can significantly enhance people’s perception of self-efficacy [34,35,36]. There exists a positive association between social support and parenting efficacy [37,38,39,40]. Those studies showed that parenting stress, social support, and parenting efficacy were closely related to each other for parents raising children with disabilities, and social support might mediate the association between parenting stress and parenting efficacy for those parents.

The aim of this study was to (a) examine the correlation between parenting stress, social support, and parenting efficacy of parents raising children with disabilities and (b) to examine the effect of social support on the association between parenting stress and parenting efficacy for those parents. Based on previous research and the unique background of parenting children with disabilities in China, we hope this study will facilitate policymakers and social workers in their assistance with families of children with disabilities and help alleviate parent stress for those families.

The following are the hypotheses examined in the study:

**Hypothesis** **1 (H1).** There is a negative correlation between parenting stress and parenting efficacy for parents of children with disabilities in China.

**Hypothesis** **2 (H2).** There is a negative correlation between parenting stress and social support for parents of children with disabilities in China.

**Hypothesis** **3 (H3).** Social support plays a mediating role between parenting stress and parenting efficacy for parents of children with disabilities in China.

## 2. Method

### 2.1. Participants and Procedure

Parents of children with disabilities participated in the study. We contacted principals of special education schools and the head of the rehabilitation institution to ask them to distribute the online parent questionnaire to parents of children with disabilities. Informed consent was presented with the link to access the questionnaire. Participation was voluntary, and participants were well informed about the aim of the study. They were also informed that they could quit at any time they wanted. The questionnaire was validly filled out by 374 parents from 374 families. The basic demographic information of the participating parents is shown in Table 1.

### 2.2. Measures

Parenting stress. Parenting stress was measured using the Chinese version of the Parental Stress Index-Short Form (PSI-SF) [41]. Specifically, PSI-SF includes three sub-scales, including parental distress (e.g., “I often feel like I can’t handle things well”), parental-child dysfunctional interaction (e.g., “my child seems to learn more slowly than other children”), and difficult child (e.g., “my child had more problems than I expected”). There are 12 items in each sub-scale utilizing a 5-point Likert scale (i.e., from 1 = strongly disagree to 5 = strongly agree). The higher the score, the greater the parenting stress. The Chinese version of the PSI-SF has been validated to its reliability and validity [42,43]. The internal consistency coefficient of the PSI-SF in the study reached an upper level (α = 0.93).

Children’s problem behavior and parents’ efficacy. The Child Adjustment and Parent Efficacy Scale-Developmental Disability (CAPES-DD), consisting of 30 items, was applied in the current study. The scale is formed by the Intensity scale and Self-Efficacy scale [44]. The Intensity scale accesses the emotional and behavioral problems of children with disabilities, and the Self-Efficacy scale measures the caregiver’s confidence in being able to cope with the emotional and behavioral problems. The Self-Efficacy scale is rated for the difficulties in dealing with children’s problem behaviors (e.g., making rude noises or saying rude words) from 1 (“Certain I can’t manage it”) to 10 (“Certain I can manage it”) over the past four weeks by the caregiver. The internal consistency coefficient of the scale was 0.90.

Social Support. The Social Support Scale, a 10-items scale, was applied in the study [45]. The scale is formed by three sub-dimensions, including subjective support (e.g., “how many close friends can you ask for help and support”), objective support (e.g., “what were the sources of financial and tangible supports you have received when you were experiencing difficult emergencies”), and support utilization (e.g., “who would you turn to when you were in trouble”). The reliability and validity evidence was collected for using the scale in previous studies [42]. The higher the score, the higher the level of social support. The internal consistency coefficient was 0.76 for this study.

### 2.3. Data Analyses

First, a descriptive statistical analysis of parenting stress, parenting efficacy, and social support was conducted using SPSS (Version 24.0). Second, their correlation was analyzed by utilizing Pearson’s correlation coefficient in SPSS. Third, the mediating effect of social support between parenting stress and parenting efficacy was examined using the AMOS (Version 27.0) [46].

## 3. Results

### 3.1. Common Method Deviation Test

Harman single-factor test was used to test the common method bias to control the common method bias. We found the first factor (without rotation) only accounted for 17.85% of the total variation and did not exceed the criterion of 40% of the total variation, indicating that there is no obvious common method bias in the data of this study.

### 3.2. Description and Correlation of Variables

Descriptive statistics and correlation analysis were conducted on parenting stress, parenting efficacy, social support, and children’s problem behavior. The mean, standard deviation, and correlation coefficients between these variables are shown in Table 2. The results indicated that parenting stress had a significant positive association with children’s problem behavior (r = 0.59, *p* < 0.01). Parenting stress was negatively correlated with parents’ efficacy (r = −0.31, *p* < 0.01). A significant negative correlation between parenting stress and social support (r = −0.30, *p* < 0.01) was found as well. Parenting efficacy was positively related to social support (r = 0.22, *p* < 0.01).

### 3.3. The Mediating Role of Social Support

**Figure 1:**
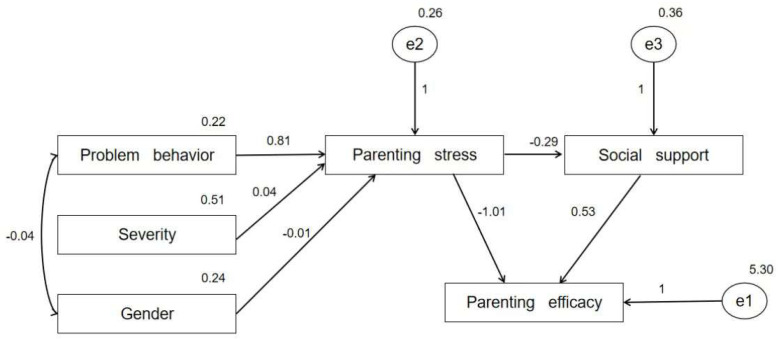
The path of the structural equation model.

To examine the internal mechanism of parenting stress, social support, and parenting efficacy, we first incorporated the variables into the structural equation model for fitting usin. Gender and severity were introduced to the model as two co-variables. According to the modification index (MI), there may be a strong correlation between the problem behavior and gender. In this study, AMOS was used to fit the above structural equation model, and the maximum likelihood method was used to estimate the model. The schematic diagrams of regression coefficients and their structural models are shown in Table 3 and Figure 1 respectively.

As indicated by the fitting results, the regression coefficient of the interaction between problem behavior and gender was −0.034, and it was significant at the 0.01 significance level. This suggested that problem behavior was more pronounced in boys than in girls. Parenting stress had a significant and direct negative influence on social support, with a coefficient of −0.291. When parenting stress was high, parents’ perception of social support decreased significantly. At the same time, parental stress had a significant direct impact on parenting efficacy, with a coefficient of −1.008, indicating that when parenting stress was high, parents had a significantly lower level of parenting efficacy. Social support also had a significant and direct positive effect on parenting efficacy, with a coefficient of 0.532. This indicates that when parents perceived a higher level of social support, they had a better sense of parenting efficacy.

The indirect effect of parenting stress on parenting efficacy was also significant, with a coefficient of −0.155. In this process, parental stress indirectly impacted parenting efficacy through its impact on social support. In summary, the negative impact of parenting stress on parenting efficacy was partly directly generated and partly indirectly generated through the mediating variable social support, in which social support was a partially mediating variable.

## 4. Discussion

The study aimed to investigate the status quo of parenting efficacy of parents having children with disabilities and to analyze the association between parenting efficacy, parenting stress, and social support. The level of self-efficacy of parents raising children with disabilities and the factors impacting parenting efficacy were not extensively examined in previous studies. Therefore, findings of the current study provided information about how parenting stress and social support were related to parenting efficacy of parents having children with disabilities.

This study found that parenting efficacy of parents having children with disabilities in China was at the medium level (6.27 ± 2.44), which was compatible with the findings of Feng et al. (2022) with a Chinese sample [47]. The findings confirmed that although parents having children with disabilities experienced higher parenting stress compared to parents with typically developing children [48], they can still develop a moderate level of parenting efficacy with social support [49,50,51]. Parenting efficacy varied across populations from different countries. In this study, parents of children with disabilities scored lower on parenting efficacy compared to the scores (8.13 ± 1.97) obtained from parents with intellectual disabilities children in Spain in Seijo et al. (2021) [52]. The lower level of parenting efficacy experienced by Chinese parents having children with disabilities might be related to the poor social support system [48], low social acceptability [53,54], stigmatization of the disabled [55], and lack of parenting knowledge and skills [56]. Previous studies found that Chinese parents of children with disabilities generally lacked a social support system, and obtained support primarily from family members due to a lack of formal support from the government or schools [48,57]. Of note, Chinese parents from Confucian cultures, living in a collective environment since childhood, may feel shame and guilt about bringing burdens to society [53], thus leading to low social acceptability [54] and severe stigmatization attached to problem behaviors [55]. Moreover, Chinese parents lacked the necessary knowledge about intervention and skills to implement intervention and faced challenges to access high-quality services [56].

We also found a negative correlation between parenting stress and parenting efficacy. This finding confirmed the findings from the previous studies [50,58,59] that reducing parenting stress might help improve parenting efficacy among parents of children with disabilities. Jandrić et al. (2021) pointed out that perceived stress can negatively predict parenting satisfaction and self-efficacy in children with or without intellectual disabilities [60]. Based on Bandura’s self-efficacy theories, individual self-efficacy might be influenced by vicarious experience, verbal persuasion, and physiological arousal [18]. Success or failure in prior parenting experiences were predictors of parenting efficacy [61]. Parents of children with disabilities suffered greater parenting stress and experienced more failure feelings, which might reduce their self-efficacy and have a negative impact on their mental health [62]. Alternatively, parents with high parenting efficacy can be more confident when facing difficulties in parenting, have more parent–children interactions, and reduce parenting stress accordingly [63].

Moreover, there was a significant negative correlation between parenting stress and social support, which aligns with findings from the previous studies [32,64]. This result suggested that parenting stress experienced by parents raising children with disabilities might decrease the possibility of gaining social support, as too much parenting stress can negatively impact parents’ ability to seek social help, or even lead to the abandonment of their children [48]. Meanwhile, previous studies have demonstrated that various forms of social support as a coping mechanism could be a buffer against stress [65,66]. Those social support, including social networks [67,68], material happiness, and family social climate [69], are related to reducing parental stress. Therefore, parents with greater social support experience were likely to have lower parenting stress.

In addition, the results indicated that there was a positive correlation between social support and parenting efficacy [70,71]. One possible reason is that social support is an instrumental aid, emotional concern, and the flow of information between people [72], which can improve psychological endurance and help parents become more capable of carrying out the difficult parenting tasks [73]. Moreover, parents with a high level of self-efficacy have stronger psychological defense mechanism [74], and more confidence in parenting, thus making full use of the social resource [75]. Therefore, improving the quality of social support is a pathway to increase parenting efficacy, particularly for parents having children with disabilities [76]. Similarly, helping parents learn effective parenting strategies and increase self-efficacy has flow-on effects on reducing psychological stress [77], which can further encourage them to gain more social support.

Finally, we tested preliminary hypotheses regarding social support, parenting stress, and parenting efficacy. This study provided evidence that social support played a mediating role in the relationship between parenting stress and parenting efficacy for parents of children with disabilities. The findings indicated that parenting stress might indirectly affect parenting efficacy through the impact of social support. Social support referred to the provision of emotional, informational, and instrumental assistance from social networks [78,79], which can relieve the stress of life to some extent. Having children with disabilities was considered a private family issue. Some parents might accept others’ support while most of them might refuse the support to avoid potential social discrimination, especially in Chinese culture [80,81]. Previous studies have indicated that Chinese families of children with disabilities, particularly in the rural community, suffered from severe discrimination in terms of rights to care and protection, economic security, developmental support, and social participation, making parents reluctant to seek support [82].

On one hand, if social support is available to parents of children with disabilities, it will provide a protective environment which brings love, care, and attachment to those families [83,84]. The social support system will also promote mental health and life satisfaction for the families during stressful times [27,85]. On the other hand, social support is also a direct source of positive emotions and a factor helping reduce negative emotions [86,87]. Individual perceived support is a psychological reality, which affects people’s behaviors [33,88]. If parents of children with disabilities actively seek social support, it will enhance their well-being, parenting quality, parenting efficacy, and child resilience [83,89]. In conclusion, social support can be provided as an intermediary, and parenting stress indirectly influenced parenting efficacy through social support for parents of children with disabilities.

## 5. Practical Implications

This study implied that parents of children with disabilities face higher parenting stress and parenting stress is negatively correlated with social support. First, we should attach great importance to the problem behaviors of children with disabilities, and provide their parents with more professional support to enhance parenting skills and confidence [79,90], as well as reduce family dysfunction and relieve parenting stress [91]. Meanwhile, this study also found parental efficacy and social support for parents of children with disabilities are positively correlated. The World Health Organization and UNICEF have suggested that the most effective way to promote children’s development is to offer nursing care [92]. Thus, professional intervention strategies should be provided for to enhance nursing care and parenting efficacy. Specifically, It is suggested to make full use of the school, community, and Internet resources to carry out targeted intervention activities, such as family focused psycho-educational therapy, online parenting programs, and targeted parenting training [93,94,95]. Moreover, the government should enact policies to offer more formal social support (including economic support, emotional support, etc.) for parents of children with disabilities, to reduce parenting stress and improve parenting efficacy.

## 6. Limitations and Further Research

There were some limitations in this study. First, the questionnaire used in this study relies on a self-reported approach to measuring parenting efficacy in a single situation. As a result, this might have been partially biased, for participants may embellish their actions. Future studies could collect this information from multiple sources. Second, it is a cross-sectional research that also limits the possibility of interpreting the directionality of the relationships. Longitudinal investigations on this topic will be useful in future studies.

## 7. Conclusions

In conclusion, this study further confirmed that parenting efficacy of Chinese parents of children with disabilities needs to be improved. Moreover, parenting stress is negatively related to parenting efficacy; parenting stress is negatively related to social support; social support is negatively correlated with the parenting efficacy for parents of children with disabilities. We also found that social support functioned as an intermediary between parenting stress and parenting efficacy, parenting stress directly and indirectly influencing parenting efficacy through social support for parents of children with disabilities. Attention should be paid to reducing parenting stress, implementing relevant interventions, and providing professional parenting training and social support for parents of children with disabilities to help improve parenting efficacy.

## Figures and Tables

**Table 1:** Demographic information of participants.

Parents’ Information (*N* = 374)		*N*	Percentage (%)
Parents	Father	126	33.7
	Mother	248	66.3
Education background	High school and below	235	62.8
	Junior college	74	19.8
	Bachelor	49	13.1
	Graduate	16	4.3
Disability	Intellectual disability	112	29.9
	Hearing disability	102	27.3
	Visual disability	31	8.3
	Autism	76	20.3
	Physical disability	4	1.1
	Cerebral palsy	13	3.5
	ADHD	6	1.6
	Multiple disabilities	30	8.0
School attendance	In school	206	55.1
	Stay at home in the school age	127	34.0
	Work	37	9.9
	Not reported	4	1.1
Age of child	0–6 years old	50	13.6
	6–12 years old	130	34.8
	12–18 years old	99	26.5
	>18 years old	95	25.1

**Table 2:** Description and correlation of variables.

	Mean ± SD	1	2	3	4
1. parenting stress	3.01 ± 0.64	1			
2. problem behavior	1.52 ± 0.47	0.59 **	1		
3. parenting efficacy	6.27 ± 2.44	−0.31 **	−0.31 **	1	
4. social support	2.74 ± 0.63	−0.30 **	−0.13 *	0.22 **	1

Note: * *p* < 0.05, ** *p* < 0.01.

**Table 3:** The outcome of model fitting.

	Estimate	S.E.	C.R.	*p*
Problem_behavior→Parenting_stress	0.807	0.056	14.305	***
severity→Parenting_stress	0.042	0.037	1.117	0.264
gender→Parenting_stress	−0.015	0.055	−0.266	0.791
Parenting_stress→Social_support	−0.291	0.049	−5.98	***
Parenting_stress→parenting_efficacy	−1.008	0.195	−5.171	***
Social_support→parenting_efficacy	0.532	0.198	2.69	0.007
Problem_behavior ↔gender	−0.034	0.012	−2.817	0.005

Note: *** *p* < 0.001.

## Data Availability

The datasets generated during and analyzed during the current study are available from the corresponding author upon reasonable request.

## References

[1] Deater-Deckard K. (2004). Parenting Stress.

[2] Abidin R.R. (1990). Introduction to the special issue: The stresses of parenting. J. Clin. Child Psychol..

[3] Baker B.L., McIntyre L.L., Blacher J., Crnic K., Edelbrock C., Low C. (2003). Pre-school children with and without developmental delay: Behavior problems and parenting stress over time. J. Intellect. Disabil. Res..

[4] Davis N.O., Carter A.S. (2008). Parenting stress in mothers and fathers of toddlers with autism spectrum disorders: Associations with child characteristics. J. Autism Dev. Disord..

[5] Glenn S., Cunningham C.C., Poole H.L., Reeves D., Weindling M. (2009). Maternal parenting stress and its correlates in families with a young child with cerebral palsy. Child Care Health Dev..

[6] Sakkalou E., Sakki H., O’reilly M.A., Salt A.T., Dale N.J. (2018). Parenting stress, anxiety, and depression in mothers with visually impaired infants: A cross-sectional and longitudinal cohort analysis. Dev. Med. Child Neurol..

[7] Jean Y.Q., Mazlan R., Ahmad M., Maamor N. (2018). Parenting Stress and Maternal Coherence: Mothers with Deaf or Hard-of-Hearing Children. Am. J. Audiol..

[8] Theule J., Wiener J., Tannock R., Jenkins J.M. (2013). Parenting Stress in Families of Children With ADHD: A Meta-Analysis. J. Emot. Behav. Disord..

[9] Crnic K., Ross E. (2017). Parenting stress and parental efficacy. Parental Stress and Early Child Development.

[10] Rao P.A., Beidel D.C. (2009). The impact of children with high-functioning autism on parental stress, sibling adjustment, and family functioning. Behav. Modif..

[11] Mak M.C.K., Yin L., Li M., Cheung R.Y., Oon P.-T. (2020). The Relation between Parenting Stress and Child Behavior Problems: Negative Parenting Styles as Mediator. J. Child Fam. Stud..

[12] Costa N.M., Weems C.F., Pellerin K., Dalton R. (2006). Parenting stress and childhood psychopathology: An examination of specificity to internalizing and externalizing symptoms. J. Psychopathol. Behav. Assess..

[13] Madarevic M., Esch L.V., Lambrechts G., Ceulemans E., Leeuwen K.V., Noens I. (2022). Parenting behaviors among mothers of pre-schoolers on the autism spectrum: Associations with parenting stress and children’s externalizing behavior problems. Res. Autism Spectr. Disord..

[14] Tomeny T.S. (2017). Parenting stress as an indirect pathway to mental health concerns among mothers of children with autism spectrum disorder. Autism.

[15] Lin Y.F., Chung H.H. (2002). Parenting stress and parents’ willingness to accept treatment in relation to behavioral problems of children with attention-deficit hyperactive disorder. J. Nurs. Res..

[16] Kazdin A.E. (1995). Child, parent and family dysfunction as predictors of outcome in cognitive-behavioral treatment of antisocial children. Behav. Res. Ther..

[17] Wolf L.C., Noh S., Fisman S.N., Speechley M. (1989). Brief report: Psychological effects of parenting stress on parents of autistic children. J. Autism Dev. Disord..

[18] Bandura A. (1977). Self-Efficacy: The Exercise of Control.

[19] Bandura A. (1989). Regulation of cognitive processes through perceived self-efficacy. Dev. Psychol..

[20] Kendall S., Bloomfield L. (2005). Developing and validating a tool to measure parenting self-efficacy. J. Adv. Nurs..

[21] Belsky J., Jaffee S.R. (2015). The multiple determinants of parenting. Developmental Psychopathology: Volume Three: Risk, Disorder, and Adaptation.

[22] Coleman P.K., Karraker K.H. (2003). Maternal self-efficacy beliefs, competence in parenting, and toddlers’ behavior and developmental status. Infant Ment. Health J..

[23] Jones T.L., Prinz R.J. (2005). Potential roles of parental self-efficacy in parent and child adjustment: A review. Clin. Psychol. Rev..

[24] Primack B.A., Hendricks K.M., Longacre M.R., Adachi-Mejia A.M., Weiss J.E., Titus L.J., Beach M.L., Dalton M.A. (2012). Parental efficacy and child behavior in a community sample of children with and without attention-deficit hyperactivity disorder (ADHD). Atten. Deficit Hyperact. Disord..

[25] Taylor S.E., Friedman H.S. (2011). Social support: A review. The Oxford Handbook of Health Psychology.

[26] Wills T.A., Clark M.S. (1991). Social support and interpersonal relationships. Prosocial Behavior.

[27] Lu M.H., Wang G.H., Lei H., Shi M.L., Zhu R., Jiang F. (2018). Social Support as Mediator and Moderator of the Relationship Between Parenting Stress and Life Satisfaction among the Chinese Parents of Children with ASD. J. Autism Dev. Disord..

[28] Quittner A.L., Glueckauf R.L., Jackson D.N. (1990). Chronic parenting stress: Moderating versus mediating effects of social support. J. Personal. Soc. Psychol..

[29] Wang Y., Huang Z., Kong F. (2020). Parenting stress and life satisfaction in mothers of children with cerebral palsy: The mediating effect of social support. J. Health Psychol..

[30] Jeong Y.G., Jeong Y.J., Bang J.A. (2013). Effect of social support on parenting stress of Korean mothers of children with cerebral palsy. J. Phys. Ther. Sci..

[31] Lederberg A.R., Golbach T. (2002). Parenting stress and social support in hearing mothers of deaf and hearing children: A longitudinal study. J. Deaf Stud. Deaf Educ..

[32] Hassall R., Rose J., McDonald J. (2005). Parenting stress in mothers of children with an intellectual disability: The effects of parental cognitions in relation to child characteristics and family support. J. Intellect. Disabil. Res..

[33] Ren J., Li X., Chen S., Chen S., Nie Y. (2020). The Influence of Factors Such as Parenting Stress and Social Support on the State Anxiety in Parents of Special Needs Children during the COVID-19 Epidemic. Front. Psychol..

[34] Coleman P.K., Karraker K.H. (1998). Self-efficacy and parenting quality: Findings and future applications. Dev. Rev..

[35] Zeiss A., Gallagher-Thompson D., Lovett S., Rose J., McKibbin C. (1999). Self-efficacy as a mediator of caregiver coping: Development and testing of an assessment model. J. Clin. Geropsychol..

[36] Haslam D.M., Pakenham K.I., Smith A. (2006). Social support and postpartum depressive symptomatology: The mediating role of maternal self-efficacy. Infant Ment. Health J..

[37] Gao L.L., Sun K., Chan S.W. (2014). Social support and parenting self-efficacy among Chinese women in the perinatal period. Midwifery.

[38] Chou J.L., Pierce K.J., Pennington L.B., Seiler R., Michael J., Mc Namara D., Zand D. (2018). Social support, family empowerment, substance use, and perceived parenting competency during pregnancy for women with substance use disorders. Subst. Use Misuse.

[39] Ortega D.M. (2002). How much support is too much? Parenting efficacy and social support. Child. Youth Serv. Rev..

[40] Suzuki S., Holloway S.D., Yamamoto Y., Mindnich J.D. (2009). Parenting Self-Efficacy and Social Support in Japan and the United States. J. Fam. Issues.

[41] Ren W. (1995). Study on the Relationship between Parental Pressure Coping Strategies and Parent-Child Relationship Satisfaction. Master’s Thesis.

[42] Guan W.J., Yan T.R., Deng M. (2015). Characteristics of parental pressure and its relationship with quality of life of parents having children with disabilities: Mediating role of social support. Psychol. Dev. Educ..

[43] Hu X., Han Z.R., Bai L., Gao M.M. (2019). The mediating role of parenting stress in the relations between parental emotion regulation and parenting behaviors in Chinese families of children with autism spectrum disorders: A dyadic analysis. J. Autism Dev. Disord..

[44] Mazzucchelli T.G., Sanders M.R., Morawska A. (2011). Child Adjustment and Parent Efficacy Scale-Developmental Disability (CAPES-DD).

[45] Xiao S.Y. (1994). Theoretical basis and research application of the social support rating scale. J. Clin. Psychiatry.

[46] Hayes A.F. (2012). PROCESS: A Versatile Computational Tool for Observed Variable Mediation, Moderation, and Conditional Process Modeling [White Paper]. http://www.afhayes.com/public/process2012.pdf.

[47] Feng Y., Zhou X., Qin X., Cai G., Lin Y., Pang Y., Chen B., Deng T., Zhang L. (2022). Parental self-efficacy and family quality of life in parents of children with autism spectrum disorder in China: The possible mediating role of social support. J. Pediatr. Nurs..

[48] Zhao M., Fu W., Ai J. (2021). The mediating role of social support in the relationship between parenting stress and resilience among Chinese parents of children with disability. J. Autism Dev. Disord..

[49] Benzies K.M., Trute B., Worthington C. (2013). Maternal self-efficacy and family adjustment in households with children with serious disability. J. Fam. Stud..

[50] Kuhn J.C., Carter A.S. (2006). Maternal self-efficacy and associated parenting cognitions among mothers of children with autism. Am. J. Orthopsychiatry.

[51] Yavuz M., Yikmis A. (2022). An Investigation of the Relationship between Parental Self-Efficacy and Marital Adjustment Levels of Parents of Disabled Individuals. Int. J. Curric. Instr..

[52] Seijo D., Tomé D., Sanmarco J., Morawska A., Fariña F. (2021). Spanish adaptation and validation of the child adjustment and parent efficacy scale. Sustainability.

[53] Wang W.C. (2016). Social Support and Parental Stress among Parents of Young Children with Autism Spectrum Disorder: An International Comparison of United States and China. Doctoral Dissertation.

[54] Lam C.S., Tsang H., Chan F., Corrigan P.W. (2006). Chinese and American perspectives on stigma. Rehabil. Educ..

[55] Li X., Lam C.B., Chung K.K.H., Leung C. (2019). Linking parents’ self-stigma to the adjustment of children with disabilities. Am. J. Orthopsychiatry.

[56] Liao Y., Dillenburger K., Hu X. (2022). Behavior analytic interventions for children with autism: Policy and practice in the United Kingdom and China. Autism.

[57] Li X.H. (2018). Effects of social support on the quality of life of parents of autistic children based on an empirical analysis of 509 parents. Popul. Soc..

[58] Heath C.L., Curtis D.F., Fan W., McPherson R. (2015). The association between parenting stress, parenting self-efficacy, and the clinical significance of child ADHD symptom change following behavior therapy. Child Psychiatry Hum. Dev..

[59] Stephenson K.G., Fenning R.M., Macklin E.A., Lu F., Norris M., Steinberg-Epstein R., Butter E.M. (2022). Child Behavior Problems and Parenting Stress in Underserved Families of Children with ASD: Investigation of Family Resources and Parenting Self-efficacy. J. Autism Dev. Disord..

[60] Jandrić S., Kurtović A. (2021). Parenting sense of competence in parents of children with and without intellectual disability. Eur. J. Psychol..

[61] Hong X., Liu Q. (2021). Parenting stress, social support and parenting self-efficacy in Chinese families: Does the number of children matter?. Early Child Dev. Care.

[62] Auriemma D.L., Ding Y., Zhang C., Rabinowitz M., Shen Y., Lantier-Galatas K. (2022). Parenting Stress in Parents of Children with Learning Disabilities: Effects of Cognitions and Coping Styles. Learn. Disabil. Res. Pract..

[63] Abarashi Z., Tahmassian K., Mazaheri M.A., Panaghi L., Mansoori N. (2014). Parental self-efficacy as a determining factor in healthy mother-child interaction: A pilot study in Iran. Iran. J. Psychiatry Behav. Sci..

[64] Cuzzocrea F., Murdaca A.M., Costa S., Filippello P., Larcan R. (2016). Parental stress, coping strategies and social support in families of children with a disability. Child Care Pract..

[65] Bailey D., Wolfe D.M., Wolfe C.R. (1994). With a little help from our friends: Social support as a source of well-being and of coping with stress. J. Soc. Soc. Welf..

[66] Hyseni Duraku Z., Hoxha L. (2018). Self-esteem, study skills, self-concept, social support, psychological distress, and coping mechanism effects on test anxiety and academic performance. Health Psychol. Open.

[67] Frey K.S., Greenberg M.T., Fewell R.R. (1989). Stress and coping among parents of handicapped children: A multidimensional approach. Am. J. Ment. Retard..

[68] Mishra S. (2020). Social networks, social capital, social support and academic success in higher education: A systematic review with a special focus on ‘underrepresented’ students. Educ. Res. Rev..

[69] Friedrich W.N., Wilturner L.T., Cohen D.S. (1985). Coping resources and parenting mentally retarded children. Am. J. Ment. Defic..

[70] Çattık M., Aksoy V. (2018). An examination of the relations among social support, self-efficacy, and life satisfaction in parents of children with developmental disabilities. Eğitim Bilim-Educ. Sci..

[71] Lai F.J. (2013). The Relationships between Parenting Stress, Child Characteristics, Parenting Self-Efficacy, and Social Support in Parents of Children with Autism in Taiwan. Ph.D. Thesis.

[72] House J. (1983). Work, Stress and Social Support.

[73] Izzo C., Weiss L., Shanahan T., Rodriguez-Brown F. (2000). Parental self-efficacy and social support as predictors of parenting practices and children’s socioemotional adjustment in Mexican immigrant families. J. Prev. Interv. Community.

[74] Rutter M. (1987). Psychosocial resilience and protective mechanisms. Am. J. Orthopsychiatry.

[75] Mouton B., Loop L., Stiévenart M., Roskam I. (2018). Confident parents for easier children: A parental self-efficacy program to improve young children’s behavior. Educ. Sci..

[76] Day J.J., Hodges J., Mazzucchelli T.G., Sofronoff K., Sanders M.R., Einfeld S., MHYPeDD Project Team (2021). Coercive parenting: Modifiable and nonmodifiable risk factors in parents of children with developmental disabilities. J. Intellect. Disabil. Res..

[77] Sanders M.R., Mazzucchelli T.G. (2013). The promotionof self-regulation through parenting interventions. Clin. Child Fam. Psychol. Rev..

[78] Cobb S. (1976). Social support as a moderator of life stress. Psychosom. Med..

[79] Miranda A., Mira A., Berenguer C., Rosello B., Baixauli I. (2019). Parenting stress in mothers of children with autism without intellectual disability. Mediation of behavioral problems and coping strategies. Front. Psychol..

[80] Zheng L., Hu T.T., Guo P.F., Zhao H.L. (2014). Social support and subjective well-being: Meta-analysis. Soc. Psychol. Sci..

[81] Shang X., Fisher K.R. (2014). Social support for mothers of children with disabilities in China. J. Soc. Serv. Res..

[82] Shang X., Fisher K.R., Xie J. (2011). Discrimination against children with disability in China. Int. J. Soc. Welf..

[83] Halstead E.J., Griffith G.M., Hastings R.P. (2018). Social support, coping, and positive perceptions as potential protective factors for the well-being of mothers of children with intellectual and developmental disabilities. Int. J. Dev. Disabil..

[84] Hobfoll S.E. (1988). The Ecology of Stress.

[85] Norris F.H., Kaniasty K. (1996). Received and perceived social support in time of stress: A test of the social support deterioration deterrence model. J. Personal. Soc. Psychol..

[86] Song J.M., Fan H.Y. (2013). Metaanalysis of the relationship between social support and subjective wellbeing. Adv. Psychol. Sci..

[87] Ye Z., Yang X., Zeng C., Wang Y., Shen Z., Li X., Lin D. (2020). Resilience, social support, and coping as mediators between COVID-19-related stressful experiences and acute stress disorder among college students in China. Appl. Psychol. Health Well-Being.

[88] Thoits P.A. (1983). Dimensions of life events that influence psychological distress: An evaluation and synthesis of the literature. Psychosoc. Stress.

[89] Armstrong M.I., Birnie-Lefcovitch S., Ungar M.T. (2005). Pathways between social support, family well being, quality of parenting, and child resilience: What we know. J. Child Fam. Stud..

[90] James Riegler L., Raj S.P., Moscato E.L., Narad M.E., Kincaid A., Wade S.L. (2020). Pilot trial of a telepsychotherapy parenting skills intervention for veteran families: Implications for managing parenting stress during COVID-19. J. Psychother. Integr..

[91] Continisio G.I., Serra N., Guillari A., Civitella M.T., Sepe A., Simeone S., Gargiulo G., Toscano S., Esposito M.R., Raia V. (2020). An investigation on parenting stress of children with cystic fibrosis. Ital. J. Pediatr..

[92] Britto P.R., Lye S.J., Proulx K., Yousafzai A.K., Matthews S.G., Vaivada T., Perez-Escamilla R., Rao N., Ip P., Fernald L.C.H. (2017). Lancet Early Childhood Development Series Steering Committee. Nurturing care: Promoting early childhood development. Lancet.

[93] Zhou Y., Yin H., Wang M., Wang J. (2019). The effect of family-focused psychoeducational therapy for autism spectrum disorder children’s parents on parenting self-efficacy and emotion. Arch. Psychiatr. Nurs..

[94] Spencer C.M., Topham G.L., King E.L. (2020). Do online parenting programs create change?: A meta-analysis. J. Fam. Psychol..

[95] Kirkpatrick B., Louw J.S., Leader G. (2019). Efficacy of parent training incorporated in behavioral sleep interventions for children with autism spectrum disorder and/or intellectual disabilities: A systematic review. Sleep Med..

